# A Scoping Review on Melasma Treatments and Their Histopathologic Correlates

**DOI:** 10.3390/dermatopathology12020013

**Published:** 2025-04-11

**Authors:** Aurore D. Zhang, Michelle Lazar, Emiliya Akhundova, Candice E. Brem, Eric J. Beltrami, Neelam A. Vashi

**Affiliations:** 1 Department of Dermatology, Boston University School of Medicine, 609 Albany St., J502, Boston, MA 02118, USA; 2 Dermatopathology Section, Department of Dermatology, Boston University School of Medicine, 609 Albany St., J300, Boston, MA 02118, USA; 3 Dermatology Institute of Boston, Boston, MA 02118, USA

**Keywords:** melasma, dermatopathology, Wood’s lamp, laser treatment, topical therapy, retinoids, quality of life, MASI, MELASQoL

## Abstract

Melasma is an incredibly common dyschromic disorder, mostly impacting women with skin of color. There are three variants of melasma based on the depth of pathologic involvement: epidermal, mixed, and dermal. While there are many treatments for melasma, there is a paucity of research on melasma treatments and their dermatopathological correlates. A scoping review was conducted of all human trials on melasma with histopathologic analysis, including 37 trials in the final analysis. Most studies were conducted on women with a Fitzpatrick skin type of III or greater. Strong histologic evidence supports the utilization of retinols/retinoids for epidermal melasma and microneedling for dermal melasma. There is a paucity of trials conducted on melasma utilizing histologic correlates, and fewer still that are comprehensive to include analyses on quality of life.

## 1. Introduction

Melasma is a common dyschromia characterized by irregular light-to-dark brown macules and patches on sun-exposed areas [1]. It poses significant cosmetic and psychological challenges to those affected, potentially leading to a reduced quality of life. The condition predominantly affects women, with a notable prevalence in individuals with Fitzpatrick skin types (FST) III to V, especially those of Asian and Hispanic descent [2]. While chronic ultraviolet (UV) exposure, hormonal influences, and genetic predisposition are considered major contributors to its development, the exact pathogenesis of melasma remains elusive [3,4]. A better understanding of its histological features and clinical presentation is essential for tailoring effective therapeutic strategies.

Melasma is primarily classified into three anatomical patterns: centrofacial, malar, and mandibular [5]. Among these, the centrofacial pattern is the most common, involving the forehead, cheeks, upper lip, nose, and chin. The malar pattern is confined to the cheeks and nose, while the mandibular pattern affects the jawline. The severity and distribution of melasma can vary widely, influenced by factors such as ethnicity, hormonal status, and environmental exposures [2]. Chronic UV exposure is a primary driver, as it stimulates melanocyte activity and induces oxidative stress and inflammation [5,6]. Hormonal factors, such as estrogen and progesterone, are thought to upregulate melanogenesis by increasing tyrosinase activity and melanosome transfer [2,5]. There is also a genetic role in the development of melasma, as several gene polymorphisms associated with increased susceptibility to melasma have been identified [2]. These factors create a cycle wherein UV-induced damage to keratinocytes and melanocytes within the basement membrane facilitates the deposition of melanin into the dermis, where melanophages become relatively resistant to clearance [2,5]. The vascular changes and inflammation associated with melasma make it challenging to achieve long-term remission [2,5]. Demographically, melasma predominantly affects women, particularly during their reproductive years [2]. This gender predisposition is attributed to hormonal influences, including the use of oral contraceptives, pregnancy, and hormone replacement therapy [2,7]. Ethnic groups with higher baseline levels of melanin, such as Asians, Hispanics, and individuals of Middle Eastern descent, are disproportionately affected [2].

**Figure 1:**
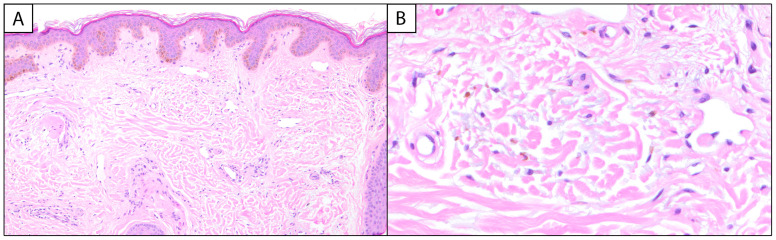
Melasma: (**A**) (100×, H&E) Histologic examination reveals variable and increased basal keratinocytic pigmentation with underlying telangiectatic blood vessels. (**B**) (400×, H&E) Higher power examination of the papillary dermis highlights pigment-laden macrophages and intermixed solar elastosis.

Melasma has unique histopathological findings, though it is often a clinical diagnosis that does not require biopsy and is characterized by epidermal hyperpigmentation with or without melanophages. Key findings include epidermal hyperpigmentation, disruption of the basement membrane, dermal melanophages, solar elastosis, and increased vascularization [2,8,9]. In the epidermis, melasma is characterized by an increased density of melanocytes and heightened melanin production [5]. Hyperactive melanocytes are often irregularly dispersed, leading to uneven pigmentation [6]. The keratinocytes in the basal and suprabasalar layers also exhibit heightened melanin uptake, contributing to the visible hyperpigmentation [5,6] (Figure 1A).

The ratio of melanocytes (MCs) to basal keratinocytes (KCs) can be increased in melasma, with a 1:1 (MC:KC) ratio reported in the literature [10]. Special stains, such as Fontana–Masson, can be employed to highlight melanin deposits within the epidermis, aiding in the differentiation of melasma from other pigmentary disorders [9]. The dermal component of melasma is equally significant. The disruption of the basement membrane, characterized by vacuolar degeneration of keratinocytes and protrusion of the basement membrane into the dermal layer, allows melanin to seep into the dermis, where it is phagocytosed by macrophages, forming dermal melanophages [6]. Melasma can be classified into three histological types based on the depth of pigment deposition: epidermal, dermal, and mixed. Epidermal melasma, the most common, involves pigment in the basal and suprabasal layers with enlarged melanocytes and prominent dendrites. Dermal melasma features pigment in both the epidermis and the upper to middle dermis, sometimes extending to the deep dermis. Mixed melasma combines both epidermal and dermal pigmentation [8]. In comparison to the epidermal type, melanophages are more commonly associated with the dermal and mixed types of melasma [6]. Additionally, solar elastosis—a hallmark of chronic sun damage—is commonly observed in the dermis (Figure 1B). This is characterized by the accumulation of abnormal elastic fibers, indicative of UV-induced skin aging [9]. Emerging evidence suggests that vascular changes play an active role in the pathogenesis of melasma. Increased vascularization, referring to the heightened presence and prominence of blood vessels in melasma-affected skin, often accompanied by the presence of mast cells, has been documented [6,9]. Mast cells are known to release pro-inflammatory mediators and angiogenic factors, further perpetuating the cycle of inflammation and hyperpigmentation [6]. Immunohistochemical staining with markers, such as CD31, can be used to assess vascular density, providing additional insights into the vascular component of melasma [11,12].

Histopathologically, melasma can be differentiated from other pigmentary disorders such as post-inflammatory hyperpigmentation (PIH), ephelides, and solar lentigines. Epidermal PIH typically presents with melanin in the basal and suprabasal epidermis, while dermal PIH is characterized by dermal melanophages and may include inflammatory cell infiltrates depending on the extent and duration of the preceding inflammation [13]. Ephelides (freckles) and solar lentigines can be differentiated histologically from melasma by differences in the characteristics of rete ridges, the dermal–epidermal junction, keratinocytes, and the number of melanocytes. These distinctions help clarify the diagnosis when clinical presentation alone is inconclusive [14].

In addition to histopathological evidence, clinical tools can be utilized to help in the diagnosis of the subtype of melasma present. Specifically, the Wood’s lamp is a valuable tool as it allows for the visualization of pigmentary changes in the skin [15,16]. Under the Wood’s lamp, providers can distinguish between epidermal and dermal melasma [15,16]. In epidermal melasma, pigmentation is enhanced under the Wood’s lamp due to the increased concentration of melanin in the superficial layers of the skin. In contrast, dermal melasma shows little to no enhancement, as the deeper melanin deposits are less visible under UV light [15,17]. Mixed melasma, which involves both epidermal and dermal components, shows partial enhancement [17]. The ability to differentiate between these subtypes is critical for guiding treatment decisions [7].

There are a myriad of treatment modalities for melasma, ranging from topical therapies to microneedling, laser treatment, and oral medications [18]. Within melasma research, there are a variety of standardized tools for scientific research, including the Melasma Severity Index (MASI) and the modified Melasma Area and Severity Index (mMASI) [19,20]. The MASI and mMASI allow for a standardized scoring of melasma, creating generalizability between studies when appropriately utilized. To ascertain the impact of melasma on quality of life, the Melasma Quality of Life Scale (MELASQoL) was created [21]. The MELASQoL assesses the psychosocial burden of melasma by evaluating factors such as embarrassment, frustration, and the effect on social and professional interactions [21]. Given its highly visible nature, melasma is known to have large impacts on quality of life (QoL), especially in more severe presentations [21].

This article seeks to further elucidate the current treatment modalities for melasma and their histologic correlates as well as the utilization of commonly used metrics in melasma research. By focusing on the histological and clinical aspects of melasma, we hope to identify effective therapeutic strategies with histological evidence. Ultimately, a deeper appreciation of the histological and clinical processes of melasma will pave the way for more targeted and effective treatments.

## 2. Materials and Methods

**Figure 2:**
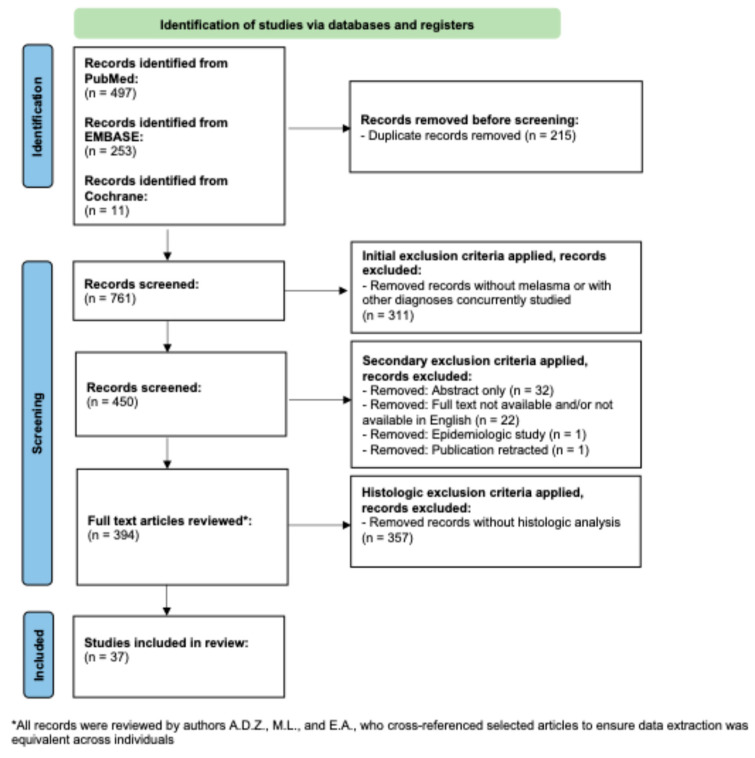
PRISMA-based flowchart for record retrieval and review, including exclusion and inclusion criteria. In total, 37 unique studies were included in this analysis.

A flow diagram of the search criteria according to PRISMA guidelines can be found in Figure 2 [22] and was registered retrospectively in OSF. A PubMed search was conducted with the term “melasma” for all clinical trials indexed in PubMed, since its initiation in December of 2024. This resulted in 497 papers. The EMBASE database was searched in December of 2024 for all clinical trials, with the term “melasma”. This yielded 253 unique studies, once duplicate entries were removed. Following this, Cochrane Reviews database was searched in December of 2024 for all entries on melasma. This yielded one systematic review, which after cross-referencing the articles derived from PubMed and EMBASE, yielded an additional 11 unique trials [23]. The total number of publications produced through this scoping search was 761.

Following this, author M.L. removed all papers without mention of melasma, trials conducted on multiple diseases concurrently, and all review papers. This yielded 450 papers. The 450 papers were then manually reviewed by authors A.D.Z., M.L., and E.A. The initial review by the authors resulted in the removal of 56 additional papers due to the full text not being available, papers not being available in English, papers being epidemiologic studies, and paper having been retracted. Therefore, after this process, the authors were left with 394 full-length unique publications of trials conducted on melasma.

Each entry was then inputted into a shared Google Sheet, and PubMed IDs were utilized to identify papers when available; otherwise, the DOI or full citation was used. Papers were reviewed in full by authors A.D.Z., M.L., and E.A. After reading the full text of the articles, authors A.D.Z., M.L., and E.A noted if the trials included a dermatopathological analysis of the effects of the treatments utilized. For the sake of this scoping review, publications were included if they involved biopsy with histological analysis, immunohistochemical analysis of the affected skin, cyanoacrylate skin surface stripping, in vivo reflectance confocal microscopy, or dermoscopic scoring of pigmentary and vascular elements of melasma. After this analysis, there were 37 studies that met the inclusion criteria [13,19,24,25,26,27,28,29,30,31,32,33,34,35,36,37,38,39,40,41,42,43,44,45,46,47,48,49,50,51,52,53,54,55,56,57,58]. All records were reviewed by authors M.L., A.D.Z., and E.A., who cross-referenced the selected articles to ensure that data extraction was equivalent across individuals.

The data extracted from the papers included number of patients enrolled, age range of the patients enrolled, gender distribution of the study population, race of the patients, Fitzpatrick skin type (FST), distribution of melasma, use of a Wood’s lamp, treatment modalities and duration, use of the MASI, efficacy of the treatments, histological analysis and findings, side effects, use of the MELASQoL, and patient preference.

## 3. Results

### 3.1. Demographic Data of the Studies

There were 37 studies included in the final analysis, published from 1994 to 2024. There was an average of 36 patients enrolled in the studies, ranging from 8 to 100 total patients enrolled. The majority of the patients enrolled were female, though four studies also included male participants [19,30,35,55]. Of the studies that commented on race, only 3 out of 13 took place on a predominantly Caucasian patient population [42,48,57]. The majority of the studies (32, 86.49%) commented on the Fitzpatrick skin type (FST) of their enrolled patient population [24,26,27,28,29,30,31,32,33,34,35,36,37,38,39,40,41,42,43,44,45,46,47,48,49,50,51,53,54,55,56,58], and most studies were conducted on individuals of FST III and IV (30, 81.08%) (Table 1) [24,26,27,28,29,30,31,32,33,34,35,36,37,38,39,40,41,42,43,44,45,46,47,48,49,50,51,53,54,55,56,58].

In eight studies, the majority of the patients had centrofacial melasma [25,30,33,38,42,43,53,57]; in seven studies, the majority had malar-pattern melasma [19,24,29,35,41,45,48]; and in no studies, did the majority of the patients had mandibular-pattern melasma. In total, 14 studies included patients with centrofacial melasma, 13 included patients with malar melasma, and 8 included patients with mandibular melasma [19,24,25,26,28,29,30,33,35,38,41,42,43,45,46,53,57,58]. The majority of the studies (21, 56.76%) utilized a Wood’s lamp to characterize the subtype of melasma present as epidermal, dermal, or mixed [13,19,24,25,27,28,29,30,37,38,42,43,45,47,49,50,51,54,55,57,58]. Fourteen studies (37.83%) had epidermal melasma as the most frequent subtype of melasma in the enrolled patients [19,24,25,26,28,30,31,38,39,43,44,46,57,58], four (10.81%) had a majority of patients with mixed melasma [29,47,49,51], and no study had a majority of patients with dermal melasma. In total, 19 studies (51.35%) included patients with epidermal melasma, 9 (24.32%) with dermal melasma, and 14 (37.84%) with mixed melasma. Only three studies required biopsy confirmation of melasma as an enrollment criterion for their study [24,44,45]; however, all included studies utilized a histological examination of treatment effects.

Of the enrolled studies, 23 (62.16%) utilized the MASI or mMASI in their study design [19,29,30,31,34,36,37,38,39,41,42,43,44,46,47,48,49,50,52,53,54,55,58]. Conversely, only four studies (10.81%) utilized the MELASQoL or a regional variant [44,45,53,54]. To assesses for patient preference, seven studies (18.92%) utilized a self-made patient satisfaction survey [32,36,37,40,50,52,55]. Fourteen studies (37.84%) utilized a mexameter, a colorimeter, and/or a spectrophotometer to measure changes in melasma over time [19,27,29,30,32,33,39,40,41,42,44,53,56,57]. Most studies utilized one to two treatment modalities (35, 94.59%), but some studies included up to four treatment modalities. The shortest study lasted 6 weeks, and the longest studies were conducted for 40 weeks. No studies included data on cost or patient ability to afford treatments.

### 3.2. Topical Treatments of Melasma

A total of 18 studies (48.65%) included topical treatments in one or more of their study arms [19,24,25,27,31,33,35,37,40,41,42,45,48,52,53,54,55,57].

Three studies utilized retinoids and/or retinols alone in one of their treatment arms [19,41,57]. Of these studies, two had a predominance of epidermal melasma and lasted between 8 weeks and 40 weeks [19,57]. Two studies showed a significant reduction in the MASI/mMASI score after treatment [19,41], and all three studies reported clinical improvement after treatment. There was no data collection regarding quality of life in these studies. Histologically, all studies utilized 2 mm to 3 mm punch biopsies collected before and after treatment. The findings included the following: decreased epidermal pigmentation (two studies), thickening of the granular layer (two studies [19,57]), increased compaction of the stratum corneum (two studies [19,57]), decreased number of melanocytes by Fontana–Masson staining (one study [41]), a significant reduction in 5-methycytosine levels in both the epidermis and the dermis (one study) [41], and a significant decrease of the expression of DNA methyltransferases (one study [41]). Common side effects reported were erythema and peeling.

Eleven studies focused on compounded lightening agents or hydroquinone alone, with five including a retinol/retinoid in combination with hydroquinone [24,25,27,31,35,37,45,48,52,53,55]. Of these studies, five had a predominance of epidermal melasma [24,25,27,35,37]. The studies lasted between 8 weeks and 24 weeks. Nine studies utilized the MASI/mMASI, and six reported statistically significant improvements in scoring after treatment [31,37,45,48,52,55]. Two studies utilized either the MELASQoL or a regional variant, both of which showed improvement after treatment [45,53]. Nine studies reported clinical improvement of melasma after treatment. Four studies utilized punch biopsies for histological analysis after treatment, five utilized in vivo reflectance confocal microscopy (RCM), one utilized corneomelametry, and one utilized advanced dermoscopic assessment. The findings included the following: decrease in epidermal pigmentation (seven studies [24,25,31,37,45,48,52]), reduction in the number of melanocytes by Fontana–Masson staining (one study [25]), reduction in melanophages and dendritic cells in the papillary dermis (one study [42]), and reduction in pseudo-network pigmentation, erythema, and telangiectasia (one study [52]). Commonly reported side effects included burning (six studies [24,25,35,37,52,55]), erythema (five studies [24,25,45,52,55]), and irritation and/or pruritus (five studies [25,31,45,52,55]).

Two studies utilized mixtures of herbal compounds [40,42]. One study was conducted solely on epidermal melasma. The duration of these studies ranged from 12 weeks to 24 weeks. They utilized the MASI/mMASI and showed significant improvement in scoring with treatment [40,42]. One study utilized a patient satisfaction survey that highlighted improved patient satisfaction scores with treatment with an herbal mixture [42]. All studies utilized RCM. The reported findings were decreased density of inflammatory cells (one study [40]), reduction in dendritic cells in the stratum spinosum (one study [40]), and decreased appearance of a hyper-refractile cobblestone pattern on RCM (one study [42]). Commonly reported side effects included erythema (one study [40]), pruritus (one study [40]), and transient xerosis (one study [42]). A summary of the reported histological findings for topical treatments of melasma can be seen in Table 2.

### 3.3. Laser Treatments of Melasma

Sixteen studies utilized laser treatments in one or more of their treatment arms [26,29,30,32,34,35,36,38,39,43,46,47,49,51,55,56]. The lasers utilized were the neodymium-doped yttrium aluminum garnet laser (Nd:YAG, eight studies [30,32,34,39,47,49,51,56]), copper bromide laser (two studies [29,35]), fractional CO_2_ laser (two studies [43,46]), 1550 nm erbium glass laser (one study [26]), Q-switched ruby laser (one study [34]), erbium-doped yttrium aluminum garnet laser (Er:YAG, one study [38]), and intense pulsed light (IPL, one study [36]). In the papers including laser treatments, the number of treatments performed ranged from 2 to 14.

The Nd:YAG laser was utilized in eight studies, of which one had a predominance of epidermal melasma, while two had a predominance of mixed melasma [30,32,34,39,47,49,51,56]. The most commonly utilized endpoint for treatment was mild erythema (five studies [32,34,47,49,51]). Five studies utilized the MASI/mMASI, and all eight studies reported improvements in the MASI/mMASI or clinical improvement of melasma with treatment. No studies utilized the MELASQoL or a variant thereof. Five studies utilized dermoscopy to assess for improvement, two utilized a confocal laser scanning microscope, and one utilized punch biopsies. The findings included reduced telangiectasia (three studies [47,49,51]), decreased density of pigmentation (two studies [49,51]), improved pseudoreticular network and globular pattern on dermoscopy (two studies [32,56]), reduction in melanin in the stratum spinosum (one study [30]), and reduced melanin granules in the cytoplasm (one study [34]). The most commonly reported side effects were erythema (four studies [32,49,51,56]), burning (one study [30]), and post-inflammatory hyperpigmentation (PIH, one study [47]).

The copper bromide laser was utilized in two studies [29,35]. Both studies utilized the MASI/mMASI and reported improvements in the scores after treatment, though in only one study it was significant. There was no commentary on quality of life. One study utilized a punch biopsy, and one utilized laser confocal microscopy for the analysis. The findings included reduction in basal pigmentation (one study [29]), reduced melanin in the basal epidermis (one study [29]), reduction in melanosomes in the epidermis (one study [29]), decrease in the number and size of blood vessels in the dermis (one study [29]), and no changes in vascularization (one study [35]). One study reported transient erythema as a side effect.

A fractional CO_2_ laser was utilized in two studies, one of which had a predominance of epidermal melasma patients enrolled [43,46]. Both studies utilized the MASI/mMASI and showed significant decreases in the scores with treatment. There were no data on quality of life or patient preference. One study utilized a punch biopsy, and one utilized advanced dermoscopic assessment. The findings included a marked decrease in pigment network (two studies [43,46]), increased epidermal thickness (one study [43]), and a decrease in the total number and size of melanocytes (one study [43]). Common side effects were erythema (two studies [43,46]) and burning (two studies [43,46]). A comparison of the findings after treatment with the most frequently studied lasers can be found in Table 3.

### 3.4. Microneedling, Injections, and Chemical Peel Treatments of Melasma

Four studies were conducted on the efficacy of microneedling, with and without other topicals, in the management of melasma [44,50,53,58]. One study contained a predominance of mixed melasma, and one epidermal melasma. The total number of treatments ranged from two to six. All four studies utilized the MASI/mMASI and showed significant improvement with treatment. Two studies used the MELASQoL or a regional variant, and both showed significant improvement with treatment [44,53]. Three studies utilized punch biopsies, and one utilized advanced dermoscopic analysis. The findings included a significant reduction in melanin density (three studies [44,53,58]), slight epidermal hyperplasia (two studies [44,53]), decreased number of pendulum melanocytes and basement membrane damage (two studies [44,53]), fibroblast proliferation (one study [44]), reduction in dermal melanophages (one study [58]), subepidermal deposition of extracellular substances (one study [44]), and reduction in total monoclonal mouse anti-melanoma antigen recognized by T cells 1 (MART-1)-positive cells (one study [58]). The most commonly reported side effects were burning (two studies [50,58]) and erythema (two studies [50,58]).

One study was conducted on the efficacy of chemical peels, one used intradermal injections of tranexamic acid without a microneedling device, and one used platelet-rich plasma injections. Two of these studies utilized the MASI/mMASI and found significant reductions in the score with treatment. One study utilized the MELASQoL and found improvement in overall quality of life. Notable findings of these studies included reduction in epidermal pigmented keratinocytes, reduction in melanophages in the upper dermis, and reduction in inflammatory infiltrate.

### 3.5. Oral Agent Treatments of Melasma

Three studies utilized oral tranexamic acid in their treatment of melasma [13,47,53]. One study was conducted solely on mixed melasma. Two studies utilized the MASI/mMASI, and one found significant improvement after treatment. Clinically, all three studies reported improvement of melasma after treatment with oral tranexamic acid [13,47,53]. One study utilized the MELASQoL and found improvement in scoring after treatment with oral tranexamic acid [53]. Histologic analysis was conducted via punch biopsy in two studies, and advanced dermoscopy in one. Notable findings included a significant decrease in epidermal pigmentation (one study [13]), reduction in melanin density (one study [47]), a significant decrease in mast cell number (one study [13]), and reduction in telangiectasia (one study [47]). Common side effects were gastritis (one study [47]) and headache (one study [53]).

## 4. Discussion

Melasma is a dyschromia that predominantly impacts women with skin of color (SOC), also defined as Fitzpatrick skin type (FST) II–VI. It is a commonly studied condition and, in some parts of the world, impacts up to 41% of the population [2]. We found that the majority of the patients enrolled in our subset of studies were patients with FST of III or greater. This is incredibly important, as it indicates that the patient population most impacted by this disorder was well represented in the studied population. This is of the utmost importance, as certain side effects, such as post-inflammatory hyperpigmentation (PIH), are much more common in SOC patients [59]. To effectively generalize research findings to the affected population, it is vital that the populations studied and the affected one be as similar as possible. Therefore, in this regard, the dermatopathological studies on melasma focus on a population that appropriately mirrors the target population.

The dyschromia characteristic of melasma is due to a variety of cellular features, such as pendulous melanocytes, which are melanocytes with elongated dendrites extending toward the dermis, that promote melanocyte migration to the dermis, increased number of inflammatory cells leading to neovascularization, and fibroblast dysregulation causing basement membrane disruption [6]. There are variants of melasma based on where the disease process is most active, i.e., epidermal, dermal, and mixed melasma. The subtype of melasma can be ascertained either by Wood’s lamp examination or by histologic analysis of tissue samples. Identifying the subtype of melasma is important, as the dermatopathology underlying the disease varies slightly based on which form of melasma is present. Certain treatments, such as hydroquinone, target melanocytes and therefore are more likely to be efficacious against epidermal melasma. Therefore, as with most conditions, the dermatopathology of melasma is directly linked to the disease process and presentation.

There is a myriad of treatment options available for melasma, including topicals, oral agents, microneedling, and even laser procedures. Additionally, there are numerous systematic reviews and care guidelines highlighting efficacious treatments for melasma [60,61,62,63,64,65]. However, even though the dermatopathology underlying various subtypes of melasma is well documented, there is no clear consensus as to which treatments lead to histological improvements. This is important to ascertain, as treatments targeting the underlying pathology are more likely to lead to increased disease-free time and overall improvements in outcomes.

Retinoids are synthetic forms of vitamin A with far-reaching effects and tools that are commonly utilized in dermatology. Topically applied retinoids reduce the transport of melanin to the epidermis and inhibit melanogenesis, leading to reduced pigmentation [66]. Within dermatology, retinoids are commonly utilized in the treatment of a variety of dyschromias, including melasma. This review highlights that retinols and/or retinoid treatments led to histological changes consistent with melasma improvement. Notable histological findings included decreased epidermal pigmentation and decreased number of melanocytes. As melanocytes are most commonly present in the epidermis, the histologic findings support the clearance of epidermal melasma but showed no evidence that supports their use against dermal melasma, whose pathology lies deeper and cannot be effectively treated with retinols/retinoids alone. Similarly, topical lightening agents often contain agents such as hydroquinone. Hydroquinone functions by inhibiting melanin synthesis by blocking tyrosinase, which is believed to mainly exert its effects in the epidermis [67,68]. Our review of the literature found that the histological changes were consistent with epidermal changes. Namely, topical lightening agents were found to decrease epidermal pigmentation and the number of melanophages present. This highlights that while commonly utilized in melasma, these treatment options will be most efficacious for patients with epidermal or mixed-type melasma. For patients with dermal melasma, the pathology lies deeper and requires different treatment modalities.

Lasers are commonly utilized in many areas of dermatologic care due to the variety of effects that they can exert based on the wavelength utilized. The most studied laser for melasma treatment with histopathological correlation is the Nd:YAG laser, with a wavelength of 1064 nm. The Nd:YAG laser is especially effective at targeting the dermis due to its longer wavelength compared to other lasers. Early histologic testing of a 1064 nm laser led to permanent leukotrichia of deep follicular cells in guinea pigs, highlighting its ability to impair dermal melanin processes [69]. The histologic findings of the studies analyzed in this review emphasized that the Nd:YAG laser led to improvement in melasma. The histologic findings were most significant for decreased pigment density and the incorporation of melanin granules into fibroblasts in the dermis. These histological findings correlated with the clinical ability of the Nd:YAG laser to treat dermal melasma and epidermal melasma, corresponding with the clinical findings reported in these trials. Other lasers were also studied, including the copper bromide laser. The copper bromide laser is a combination of yellow-green light at 578 nm and 511 nm and is most useful in the treatment of vascular lesions, as the wavelengths produced are selectively more absorbed by hemoglobin than by melanin [70]. The histological findings highlighted in this review corresponded with the hemoglobin targeting, with a reduction in the number and size of blood vessels in the dermis noted across studies. Therefore, the copper bromide laser may be best utilized in patients with severe telangiectasia or vascular components in their melasma. Utilizing a Wood’s lamp clinically or utilizing dermatopathological analysis would allow providers to identify what subtype of melasma is present and could reduce the administration of unnecessary treatments. Furthermore, doing so would allow for tailored treatments based on specific melasma presentations.

As previously mentioned, it is important to remember that most patients with melasma are SOC patients. In this review, we identified PIH as a side effect, especially with laser treatment. This is important to consider, as providers ought to practice caution when treating melasma and other dyschromic disorders in SOC populations, so not to replace the underlying dyschromia with PIH. Generally, laser treatments are safe and efficacious in skin of color patients if they are performed by trained professionals [71,72].

Injections and chemical peels are also commonly utilized in the treatment of melasma. Microneedling generates a dermal injury allowing for dermal remodeling [73]. The ingenuity of microneedling is that it creates a pathway into the dermis by which medications can be introduced, bypassing the epidermis. However, due to the damage that microneedling generates, it can also lead to epidermal changes. This review highlights that microneedling and dermal injections were found to be efficacious against epidermal, mixed, and dermal melasma. Significant histologic findings included pendulous melanocyte and basement membrane damage and a reduction in dermal melanophages. These findings explain the mechanism by which microneedling is effective in dermal melasma. Additionally, some studies highlighted a reduction in the density of epidermal melanin as well, which explains why microneedling can also be efficacious in epidermal melasma. Furthermore, there was histologic evidence of the subepidermal deposition of extracellular substances, proving that microneedling can be used to deliver medications to the dermis. All these factors in combination highlight why microneedling works for a variety of melasma subtypes and the importance of considering the dermatopathology underlying the disorder.

Several limitations in the literature were found with this scoping review. Namely, while there is a plethora of studies focusing on melasma, there is a paucity of studies conducted with a prominent histologic or dermoscopic assessment of the lesions. This is likely due to the cosmetically sensitive nature of the disorder, and many studies cited this as rationale for not including histologic samples in their study design. However, there are now many tools that allow for a near-histologic analysis of samples, such as RCM, without leading to cosmetic concerns. Another finding of this review is that while the MASI and mMASI are well-validated and utilized tools, many studies continue to omit them. For the sake of generalizability, researchers ought to include standardized metrics to increase comparability across studies. Finally, studies that did include histologic data did not include a commentary on the impact of melasma on quality of life. There is a need for comprehensive research that considers all aspects of melasma and its treatment to ensure that both clinical clearance and patient satisfaction are achieved.

## 5. Conclusions

There are a variety of treatments that can be employed in the treatment of melasma. However, the subtype of melasma should be considered in order to utilize the most efficacious treatment modalities. Retinoids are effective in the treatment of epidermal melasma, with decreased epidermal pigmentation and a decreased number of melanocytes found on dermatopathological analysis. The Nd:YAG laser was successful in the treatment of dermal melasma, with decreased pigment density and incorporation of melanin granules into fibroblasts in the dermis appreciated on histologic analysis. Future research ought to focus on treatments of melasma as well as on their histopathological correlates to ensure the longest possible efficacy of the treatments in patients. Furthermore, treatments ought to be targeted toward the specific variant of melasma present, namely, epidermal or dermal melasma, which requires a strong understanding of the dermatopathology underlying melasma. There is a need for comprehensive research that focuses on the impact of treatments on improving melasma-related quality of life dilemmas that many patients face. Additionally, research regarding the cost of the treatments and cost efficacy ought to be conducted, also ensuring that patients’ financial constraints are taken into consideration, to provide the most efficacious and affordable care to all patients.

## Figures and Tables

**Table 1:** Fitzpatrick skin type-based patient enrollment in included studies.

Fitzpatrick Skin Type (FST)	Number of Studies with FST Enrolled (%)
Fitzpatrick Skin Type I	2 (5.41%)
Fitzpatrick Skin Type II	7 (18.92%)
Fitzpatrick Skin Type III	30 (81.08%)
Fitzpatrick Skin Type IV	30 (81.08%)
Fitzpatrick Skin Type V	12 (32.43%)
Fitzpatrick Skin Type VI	2 (5.41%)

**Table 2:** Topical treatments of melasma with histological findings.

Topical Agent	Histological Findings	Side Effects
1. Retinoids and/or retinols	1. Decreased epidermal pigmentation 1. Decreased number of melanocytes by Fontana–Masson staining 1. Significant reduction in 5-methycytosine in epidermis and dermis 1. Significant decrease in the expression of DNA methyltransferases	Erythema and peeling
2. Topical lightening agents	2. Decreased epidermal pigmentation 2. Reduced number of melanophages and dendritic cells 2. Reduction in pseudo-network pigmentation	Burning, erythema, and pruritus and/or irritation
3. Herbal agents	3. Decreased density of inflammatory cells 3. Reduction in inflammatory cells in the stratum spinosum 3. Decreased appearance of hyper-refractile cobblestone patterning	Erythema, pruritus, and transient xerosis

**Table 3:** Selected laser treatments of melasma with histological findings.

Type of Laser	Histological Findings	Side Effects
1. Nd:YAG	1. Reduced telangiectasia 1. Decreased pigment density 1. Improved pseudoreticular and globular pattern 1. Reduction in melanin in the stratum spinosum 1. Incorporation of melanin granules into fibroblasts in the dermis	Erythema, burning, and PIH
2. Copper bromide	2. Reduction in basal pigmentation 2. Reduced melanin in the basal epidermis 2. Reduction in epidermal melanosomes 2. Reduction in number and size of blood vessels in the dermis 2. No change in vascularization	Erythema
3. CO_2_	3. Decrease in pigment network 3. Increased epidermal thickness 3. Decrease in total number and size of melanocytes 3. Decrease in melanin granules in melanocytes and keratinocytes	Erythema and burning

## Abbreviations

The following abbreviations are used in this manuscript:

FST	Fitzpatrick skin type
UV	Ultraviolet
MASI	Melasma Severity Index
mMASI	Modified Melasma Area and Severity Index
MELASQoL	Melasma Quality of Life scale
RCM	In vivo reflectance confocal microscopy
Nd:YAG	Neodymium-doped yttrium aluminum garnet
PIH	Post-inflammatory hyperpigmentation
